# Thickness measurements taken with the spectralis OCT increase with decreasing signal strength

**DOI:** 10.1186/s12886-022-02356-4

**Published:** 2022-04-01

**Authors:** Assaf Gershoni, Edward Barayev, Igor Vainer, Raviv Allon, Roy Yavnieli, Yinon Shapira, Michael Mimouni, Noa Geffen, Arie Yehuda Nemet, Ori Segal

**Affiliations:** 1 Department of Ophthalmology, Rabin Medical Center, Petah Tikva, Israel; 2 Sackler Faculty of Medicine, Tel Aviv University, Tel Aviv, Israel; 3 Department of Otolaryngology, Rabin Medical Center, Petah Tikva, Israel; 4 Department of Otolaryngology, Kaplan Medical Center, Rehovot, Israel; 5 Department of Ophthalmology, Meir Medical Center, Kfar Saba, Israel; 6 Department of Ophthalmology, Rambam Health Care Campus, Haifa, Israel

**Keywords:** Spectralis, Optical Coherence tomography, Macular thickness, Retinal nerve fiber layer, Signal strength

## Abstract

**Background:**

Optical coherence tomography (OCT) is used worldwide by clinicians to evaluate macular and retinal nerve fiber layer (RNFL) characteristics. It is frequently utilized to assess disease severity, progression and efficacy of treatment, and therefore must be reliable and reproducible.

**Objective:**

To examine the influence of signal strength on macular thickness parameters, macular volume measurement and RNFL thickness measured by spectral-domain optical coherence tomography (SD-OCT).

**Methods:**

Macular thickness parameters, macular volume measurement and RNFL thickness were measured by the Spectralis® OCT (Heidelberg Engineering, Heidelberg, Germany). In each eye, the focusing knob was adjusted to obtain 4 images with different signal strengths – Low (below 15), Moderate (15-20), Good (20-25) and Excellent (above 25). The relationship between signal strength and measured data was assessed using the mixed model procedure.

**Results:**

A total of 71 eyes of 41 healthy subjects were included. Central macular thickness, macular volume and mean RNFL thickness increased with decreasing signal strength. Specifically, eyes with excellent signal strength showed significantly thinner central macular thickness (*p* = 0.023), macular volume (*p* = 0.047), and mean RNFL thickness (*p* = 0.0139).

**Conclusions:**

Higher signal strength is associated with lower macular thickness, macular volume and RNFL thickness measurements. The mean differences between excellent and low-quality measurements were small implicating that SD-OCT is a reliable imaging tool even at low quality scans. It is imperative that the physician compares the signal strength of all scans, as minute differences may alter results.

## Introduction

The Optical coherence tomography (OCT) is a non-invasive imaging test used worldwide by clinicians to evaluate macular and retinal nerve fiber layer (RNFL) characteristics, which was first introduced by Huang et al. in 1991 [1]. It is an imperative tool the ophthalmologist utilizes in order to assess disease severity, progression and efficacy of treatment. However, in order to do so, OCT scans must be reliable and reproducible [2, 3].

Several studies have reported the effect of image quality on the reproducibility and quantitative measurements of OCT scans in Cirrus [4], stratus [5], and RTVue [6], mostly emphasizing the importance of high image quality for acquirement of consistent and reliable measurements. Previous studies found that signal strength was positively correlated with thickness and volume measurements when using Stratus OCT for measuring macular [7, 8] and RNFL [9, 10] parameters. A similar correlation was shown when using Cirrus OCT. [11]

The Spectralis imaging platform (Heidelberg Engineering, Germany) is a spectral-domain-OCT employed frequently throughout the world, and is one of a few OCT devices approved for international studies. The image quality of the OCT scans in the Spectralis platform is expressed by the “Q score” which is a term for signal strength. The manufacturer signal index threshold provided by Heidelberg Engineering is a Q score of 15 [12]. Strampe et al. [13] have assessed Q score effect on RNFL measurements using Spectralis OCT, and reported increasing thickness with decreasing signal strength. In the aforementioned study macular thickness and volume were not assessed.

The purpose of our study was to assess the effect of the Q score on Spectralis OCT macular thickness, macular volume and RNFL thickness measurements in healthy subjects.

## Materials and methods

This prospective cohort study followed the tenets of the Declaration of Helsinki and was approved by the Institutional Review Board of the Meir Medical Center. Written informed consent for participation in the study was obtained from all participants.

### Study cohort

The study group consisted of 82 eyes of 41 healthy individuals, which underwent multiple consecutive OCT scans between December 2015 and December 2017 at the Department of Ophthalmology, Meir Medical Center, Kfar-Saba, Israel. Subjects were included in this study if they were 18 years or older, had no history or evidence of ocular pathology or surgery, had a spherical equivalent (SE) between −6.00 and + 6.00 diopters (D), had 20/30 best-corrected visual acuity or better and had an optic nerve head and retina without abnormalities on dilated fundus examination in the 4 months preceding the OCT image acquisitions. Subjects were excluded if they had any inadequate layer segmentation in macular or RNFL scans or if media opacities precluding OCT imaging were present.

### Study procedure

Demographic data were recorded prior to examination. Subjects who met the study criteria underwent multiple, consecutive scans using the “fast macular volume” and “RNFL optic disc” acquisition protocols of the Spectralis imaging system (Version 6.7.21.0). Subjects were not pharmacologically dilated for image acquisition.

The Spectralis Q score is measured on a scale of 0-40, with 40 representing the best image quality. Varied Q scores were acquired via adjustment of the focusing knob, starting with each subjects’ individual SE, and then defocusing gradually in order to reduce the image quality, using previously described methods [7, 9]. The Q score for macular thickness and volume was calculated by averaging all Q scores from individual macular scans. Subsequently, Q scores were arbitrarily divided into 4 groups: Low (Q < 15), Moderate (15 ≤ Q < 20), Good (20 ≤ Q ≤ 25) and Excellent (Q > 25). At least one image of each eye was obtained for each Q group score. The macular OCT parameters included in the analysis were volume measurements in the 6-mm area and thickness measurements in the central 1-mm and in each quadrant in the 3-mm area (Superior, Inferior, Nasal and Temporal). All of the aforementioned measurements were obtained from the thickness map, and we extracted the corresponding Q score by retrieving the score from each frame and calculating an average Q score.

The RNFL parameters included in the analysis were thickness measurements of mean global thickness and in each of the six sectoral measurements comprising it (Temporal-superior, Temporal, Temporal-inferior, Nasal-inferior, Nasal and Nasal-superior).

### Statistical analysis

The statistical analysis for this paper was generated using SAS Software, Version 9.4. Continuous variables were presented by Mean ± Std, Categorical variables were presented by (N,%). Correlation between signal strength, thickness and volume measurements were analyzed using the Pearson correlation test. Bland-Altman analysis was used, setting the limits of agreement (LOA) to 2 standard deviations. Data was converted to a Long format, so that each data line presented results for specific subject*imaging (1-4)*Eye (Left or Right). Repeated Measure Analysis, using a Mixed Linear Model with a Compound Symmetry Covariance Structure, was used to regress imaging results on imaging number, eye, gender and age in order to deal with multiple measurements for each subject and eye. The Bonferroni correction was used for multiple comparisons of groups in the model. Two-sided p values less than .05 were considered statistically significant.

## Results

Out of eighty-two eyes of 41 participants, 71 eyes qualified for inclusion in the study. Ten eyes were excluded from the study due to segmentation errors and one eye excluded due to motion artifacts. Subject ages ranged from 18 to 68 years (median, 46.5 years). The study group consisted of 22 females (53.7%) and 19 males (46.3%). Of the 71 eyes analyzed, 36 were left ones (50.7%).

### Macular thickness

**Fig. 1:**
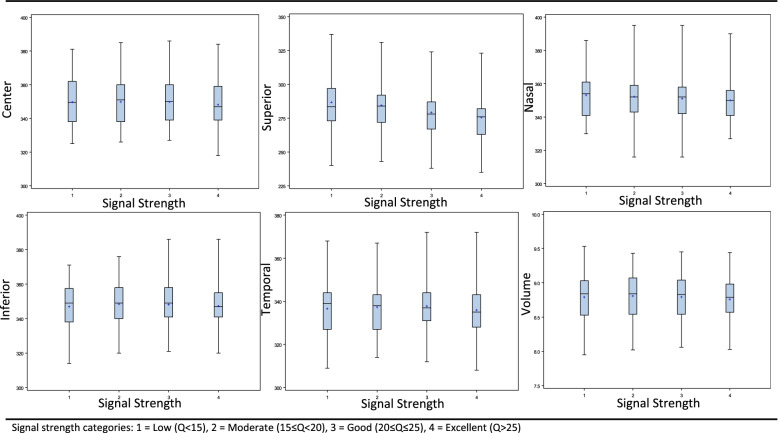
Macular Thickness and Volume measurements at different Signal Strength categories

Figure 1 displays the relationship between macular thickness and the Q score groups for each area examined. Central macular thickness measurements were lowest in the excellent quality group (Q > 25), compared with each of the other groups, all of which were statistically significant (*p* = 0.023), (Table 1). In the superior quadrant, thickness in the excellent signal strength group was lower compared with each of the other groups (*p* = 0.046). In the Nasal quadrant, thickness in the excellent signal strength group was lower compared with both the low and moderate signal strength groups (*P* < 0.001). Also, mean thickness in the good signal strength group was lower compared with the low group (*p* = 0.0095).

**Table 1:** Macular and RNFL measurements by OCT Q score category

	Low	Moderate	Good	Excellent	Difference^a^ (%)
**Macular Parameters**					
Central Macular Thickness, μm	286.73 ± 21.44	284.48 ± 19.38	†*279.21 ± 19.83	‡†*275.59 ± 19.08	−3.88
Macular Volume, mm^3^	8.79 ± 0.39	8.81 ± 0.36	8.79 ± 0.34	‡†*8.76 ± 0.33	−0.35
Inner Macular Thickness by quadrant, μm					
Superior	349.71 ± 13.84	349.83 ± 13.94	± 13.47349.77	‡†*348.18 ± 13.93	−0.44
Inferior	346.98 ± 14.48	348.44 ± 13.17	348.18 ± 13.03	347.31 ± 12.96	0.1
Temporal	336.57 ± 14.10	12.77 ± 337.39	337.85 ± 12.77	335.97 ± 13.06	−0.18
Nasal	353.18 ± 13.67	352.24 ± 14.40	*351.07 ± 14.61	†* 13.69 ± 350.1	−0.88
**Retinal Nerve Fiber Layer Parameters**					
Mean RNFL, μm	101.4 ± 10.99	100.85 ± 10.4	100.45 ± 10.4	†*99.68 ± 10.37	−1.7
RNFL by quadrant, μm					
Nasal	77.34 ± 17.31	77.2 ± 16.27	76.41 ± 16.47	76.41 ± 16.77	−1.2
Inferior-Nasal	117.89 ± 28.86	117.63 ± 28.73	117.2 ± 29.42	116.2 ± 28.89	−1.43
Inferior-Temporal	148.08 ± 19.92	147.89 ± 20.36	147.99 ± 20.64	147.00 ± 21.53	−0.73
Temporal	71.60 ± 10.96	71.42 ± 10.88	70.77 ± 10.91	70.08 ± 10.94	−2.12
Superior-Temporal	135.40 ± 17.78	134.93 ± 17.12	133.97 ± 16.44	132.38 ± 16.09	−2.23
Superior-Nasal	111.60 ± 24.63	109.76 ± 24.71	109.55 ± 24.19	108.15 ± 23.81	−3.09

Data are shown as mean ± SD

Scans were categorized according to signal strength. Low (Q < 15), Moderate (15 ≤ Q < 20), Good (20 ≤ Q ≤ 25), Excellent (Q > 25)

*RNFL* Retinal Nerve Fiber Layer, *OCT* Optical Coherence Tomography

^a^Difference is calculated between excellent and low groups, as a percentage of the low group

*Statistically significant difference, *p* < 0.05, compared with the low group

† Statistically significant difference, *p* < 0.05, compared with the moderate group

‡Statistically significant difference, *p* < 0.05, compared with the good group

### Macular volume

Macular volume measurements demonstrated the same statistically significant trait, as the excellent quality group displayed the lowest mean volume (*p* = 0.047, Table 1**,** Fig. 1).

### RNFL thickness

**Fig. 2:**
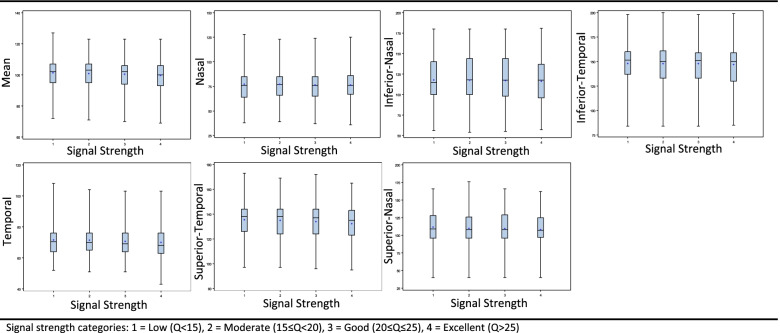
Retinal Nerve Fiber Layer Thickness measurements at different Signal Strength categories

As depicted in Table 1, RNFL thickness was lower in the excellent signal strength group than other groups, in both mean RNFL thickness and in each of the six sectoral measurements comprising it (Temporal-superior, Temporal, Temporal-inferior, Nasal-inferior, Nasal and Nasal-superior, Fig. 2). However, statistical significance was shown only for mean RNFL thickness; thickness in the excellent signal strength group was lower compared with both the low and moderate signal strength groups (*P* = 0.0139).

**Fig. 3:**
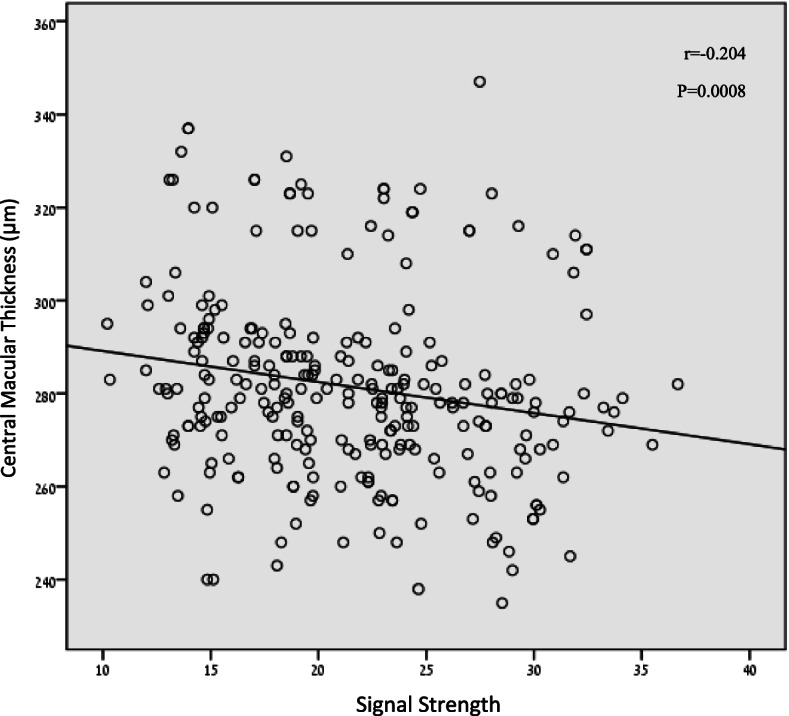
Correlation between signal strength and central macular thickness. Thickness measurement increase with decreasing signal strength. *r* = Pearson correlation coefficient

**Fig. 4:**
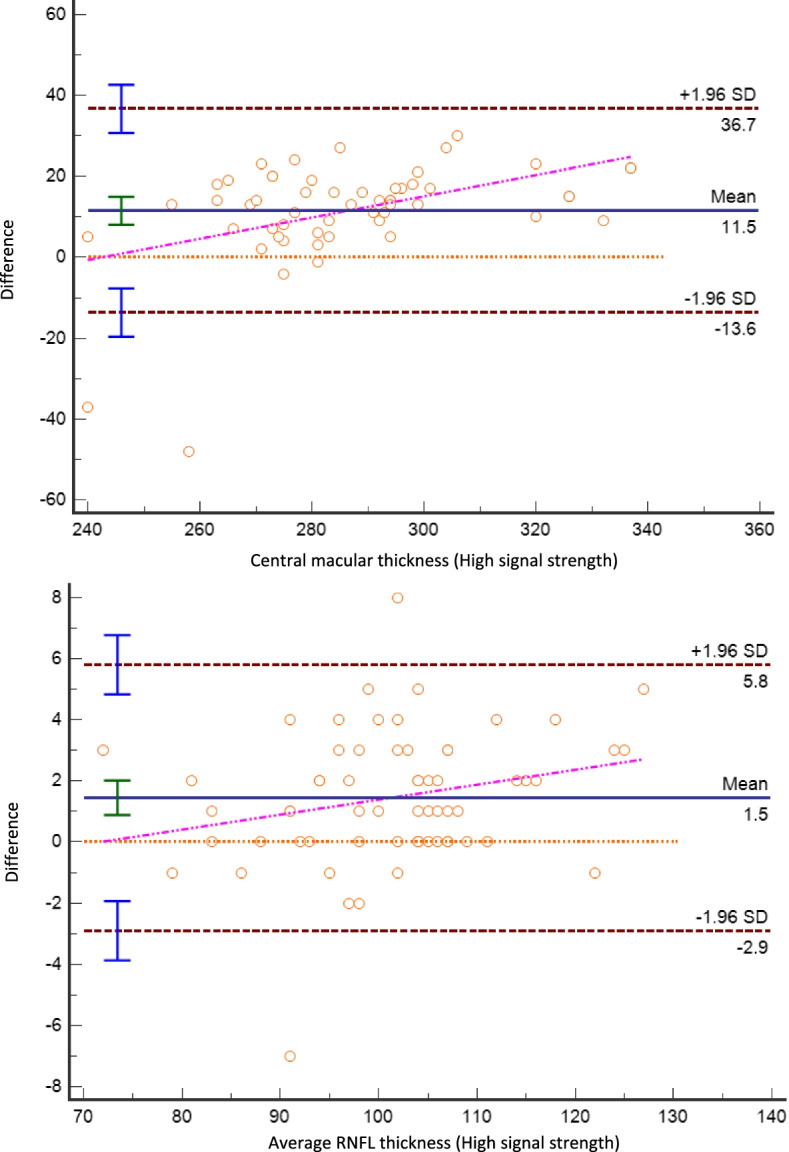
Bland-Altman analysis to test for the differences in macular (top) and retinal nerve fiber layer (RNFL) thickness measurements between excellent (>25) and low (<15). In both measurements, dispersion increases with retinal thickness

A negative correlation was found between signal strength and central macular thickness (*r* = −0.204, *p* = 0.0008, Fig. 3). No other correlations were found between thickness or volume measurements and signal strength (Table 2). A bland-Altman plot showing the differences between bad and high-quality images for both macular and RNFL thickness measurements is shown in Fig. 4.

**Table 2:** Correlations between signal strength and macular / RNFL measurements

	r	***P***
**Macular Parameters**		
Central Macular Thickness, μm	−0.204	**0.0008**
Macular Volume, mm^3^	−0.009	0.873
Macular Thickness by quadrant, μm		
Superior	−0.01	0.867
Inferior	0.034	0.572
Temporal	0.0002	0.996
Nasal	−0.05	0.407
**RNFL Parameters**		
Mean RNFL, μm	−0.058	0.331
RNFL by quadrant, μm		
Nasal	−0.021	0.727
Inferior-Nasal	−0.031	0.612
Inferior-Temporal	−0.039	0.517
Temporal	−0.062	0.303
Superior-Temporal	−0.039	0.518
Superior-Nasal	−0.033	0.588

*RNFL* Retinal Nerve Fiber Layer

*r* = Pearson correlation coefficient

*P* values in bold indicate significant associations

## Discussion

Modern OCT allows noninvasive high-resolution imaging of various retinal pathologies. The Spectralis OCT is utilized worldwide to diagnose and monitor macular and optic disc diseases [14, 15], but in order for the physician to make clinical decisions which are based on it, the information gathered must be reliable and reproducible. However, the Spectralis OCT’s image quality, which is expressed by the Q score, can vary due to conditions such as media opacities or low visual acuity [16, 17]. A Q score of at least 15 is recommended by the manufacturer [12, 18], not specifically for retinal disease.

We found that macular thickness, macular volume and mean RNFL thickness information obtained from Spectralis OCT scans with an average Q score > 25 are lower than in scans with lower Q scores. A Pearson correlation between signal strength and central macular thickness also shows a negative correlation, meaning that thickness measurements decrease with increasing Signal Strength.

Although much of our results were statistically significant, it is important to note that the biggest average difference measured was under 4%. These changes are usually of no clinical significance, and within the acceptable margin of error both for RNFL and macular measurements. However, in some cases, like severe thinning of the RNFL, these changes can be of clinical importance [19]. In addition, when examining patients over time, even small changes can be of significance [20]. Lastly, as can be seen in the Bland-Altman plot (Fig. 4), although the average differences were small, in some cases thickness measurements differences between bad and high-quality central macular images were as high as 36 μm, which could bear significant clinical relevance. These differences also tended to be higher with higher macular thickness.

To the best of our knowledge, this is the first report to comprehensively investigate the effect of Q scores on macular thickness and volume measurements in healthy individuals in the Spectralis OCT device. Several studies have assessed the potential effect of signal strength on RNFL and macular thickness measurements using the stratus [7–9] and cirrus [11] OCT. These studies have found that thickness measurements increased with increasing signal strength, as can be seen in Table 3. There are several possible explanations for the influence of low-quality scans on RNFL and retinal measurements. Increased noise in lower quality images may cause segmentation algorithms to inaccurately identify retinal layers. Different machines use different segmentation algorithms, so noise might affect segmentation differently between machines, as both Spectralis and Cirrus machines use spectral domain technology, but have different influence of signal strength on thickness measurements [21]. Perhaps there is also some influence to the fact that different machines use different segmentation of the outer border of the retina (Table 3) [22]. In the current study we meticulously assessed all images to verify that segmentation was correct prior to analysis.

**Table 3:** comparison of the previous studies for other OCT devices

	Current study	Kim et al. [4]	Zhang et al. [6]	Wu et al. [5]
**OCT device tested**	Spectralis	Cirrus	RTVue	Stratus
Acquisition technology	Spectral domain	Spectral domain	Fourier domain	Time domain
Scan speed (A-scans per second)	40,000	27,000	26,000	768
Axial resolution (μm)	7	5	5	10
Transverse resolution (μm)	14	20	15	20
Quality score scale	(Q, 0-40)	(SS, 0-10)	(SSI, 0-100)	(SS, 0-10)
Minimal suggested quality by manufacturer	15	6	30	6
Quality score & thickness relationship	Thickness decreases with higher SS (RNFL & macula)	Thickness increases with higher SS (RNFL)	Thickness increases with higher SS (RNFL)	Thickness increases with higher SS (RNFL)
Outer retina segmentation border	outer reflective band	outer reflective band	second inner hyperreflective band	inner hyperreflective band

*OCT* Optical Coherence Tomography, *RNFL* Retinal Nerve Fiber Layer, *SS* Signal strength, *SSI* Signal Strength Index

In 2009, Balasubramanian et al. [21] suggested that retinal thickness measurements with the Spectralis OCT increased as a function of decreasing SS, but statistically validated results were not available as the study only included 4 individuals. Strampe et al. [13] analyzed these changes using the Spectralis OCT and found a statistically significant linear relationship between Q score and RNFL thickness. However, their study only included 30 eyes and only showed RNFL thickness measurements. Our Study provides, for the first time, important information regarding macular thickness and volume measurements. In addition, we measured Q for macular thickness and volume by averaging all the macular scans. Perhaps in the future this process may be simplified by automatic measurements with automated software correcting measurements based on quality of the images.

As we only included scans with correct segmentation, differences between scans were small even at low signal strengths and did not reach clinical difference, although reaching statistical significance. As most studies regarding Spectralis OCT only include scans with Q score above 15 [12, 23], 20 [24], or even 25 [25], our study shows for the first time that the Spectralis OCT could be considered as a reliable tool even at Q scores below 15, if correct segmentation is confirmed.

This study has several limitations. First of which is its relatively small sample size. Second, our study was conducted on eyes of healthy individuals with no ocular disease. Thus, our conclusions may not apply when examining eyes with pathology. Third, subjects were not pharmacologically dilated for image acquisition so there is the possibility that the results may be different in eyes with pupil dilation, although it has been reported that dilation does not affect measurements with SD-OCT. [26] Lastly, using both eyes from the same subject for almost all the sample size is also an important limitation, as some factors of those subjects may influence in both images, in spite of the statistical ways we used to address this.

In conclusion, to the best of our knowledge, this is the first study to assess the effect of Q value on macular thickness and volume measurements with Spectralis OCT. Higher signal strength leads to lower thickness for both the macula and RNFL measurements in most examined areas. This difference is not clinically significant, implicating that the Spectralis OCT is a reliable imaging tool even at low quality scans. Nevertheless, upon examining follow-up scans of the same patient, it is imperative that the physician compares the signal strength of all scans, as minute differences may alter results.

## Data Availability

All data relevant to the study are included in the article or uploaded as supplementary information. All data were included in the article and its associated supplementary materials and open to public.
